# SPLUNC1/BPIFA1 Restrains Neutrophil-Dominant Inflammation and Coordinated Inflammatory Gene Programs During LPS-Induced Lung Injury

**DOI:** 10.3390/biom16091349

**Published:** 2026-09-16

**Authors:** Hexin Lu, Yuanpu Peter Di

**Affiliations:** 1 Department of Environmental and Occupational Health, University of Pittsburgh, Pittsburgh, PA 15260, USA; hel102@pitt.edu; 2 Department of Cellular & Molecular Medicine, Center for Translational Science, Herbert Wertheim College of Medicine, Florida International University, Port St. Lucie, FL 34987, USA

**Keywords:** SPLUNC1/BPIFA1, lipopolysaccharide, inflammation, acute lung injury, airway innate immunity

## Abstract

Acute lung injury (ALI) is characterized by alveolar-capillary barrier disruption, increased pulmonary permeability, impaired gas exchange, and inflammation. Lipopolysaccharide (LPS), a potent microbial trigger of inflammation, is widely used to model ALI. Short palate, lung, and nasal epithelial clone 1 (SPLUNC1), also known as BPIFA1, is an abundant surfactant-like protein secreted by the airway epithelium with antimicrobial and immunomodulatory functions. However, its role in endotoxin-induced lung injury remains incompletely understood. We compared pulmonary responses to LPS in wild-type and SPLUNC1/BPIFA1-knockout mice. Mice received intranasal PBS or 5 μg LPS were evaluated 24 h later by bronchoalveolar lavage, histology, cytospin, flow cytometry, qPCR, ELISA, and whole-lung RNA sequencing. Compared with WT mice, LPS-challenged KO mice exhibited greater inflammatory-cell accumulation, enhanced neutrophil recruitment, increased cytokine and chemokine expression, and greater histologic lung injury. Transcriptomic analyses showed preferential activation of TNFα/NF-κB, IL-6-JAK-STAT3, complement, cytokine/chemokine, myeloid/neutrophil, and stress-response in KO lungs. These findings identify SPLUNC1/BPIFA1 as an epithelial-derived regulator that restrains endotoxin-induced inflammatory signaling, neutrophil recruitment, and tissue injury. Collectively, these results support a protective role for SPLUNC1/BPIFA1 in acute pulmonary inflammation and provide a rationale for investigating SPLUNC1/BPIFA1 augmentation to mitigate ALI. Further studies evaluating SPLUNC1-based interventions in preclinical models are warranted.

## 1. Introduction

Acute lung injury (ALI) is an acute inflammatory process characterized by disruption of the alveolar-capillary barrier, increased pulmonary permeability, inflammatory-cell accumulation, and impaired gas exchange [1]. In its clinically recognized form, this process underlies acute respiratory distress syndrome (ARDS), a life-threatening cause of hypoxemic respiratory failure arising from direct pulmonary insults, such as pneumonia and aspiration, or indirect systemic insults, including sepsis and major trauma [2,3,4,5]. Although advances in supportive care have improved outcomes, effective pharmacologic therapies that directly modify the inflammatory and tissue-injury processes remain limited. A better understanding of the endogenous mechanisms that regulate pulmonary inflammation is therefore essential for identifying new strategies to prevent or attenuate ALI.

Lipopolysaccharide (LPS) is a major component of the outer membrane of Gram-negative bacteria, which is one of the most potent microbial activators of innate immunity and is widely used to model ALI experimentally [6,7,8,9]. LPS is an endotoxin and a potent activator of the immune system, interacting with Toll-like receptor 4 (TLR4) on immune cells [9,10]. This interaction triggers signaling events by activating MyD88- and TRIF-dependent pathways and downstream nuclear factor κB (NF-κB) and promoting the production of pro-inflammatory cytokines and chemokines, such as tumor necrosis factor-α (TNF-α), interleukin-6 (IL-6), and interleukin-1β (IL-1β) [11,12,13,14]. Neutrophils are particularly important during the early response to LPS. Although their recruitment is required for antimicrobial defense, excessive or dysregulated neutrophil activation can damage the epithelial and endothelial barriers through the release of proteases, reactive oxygen species, inflammatory mediators, and extracellular traps [15,16]. The magnitude and consequences of the pulmonary response to LPS are therefore determined not only by pathogen-recognition pathways but also by endogenous airway factors that regulate inflammatory-cell recruitment and activation [16,17,18].

Short palate, lung, and nasal epithelial clone 1 (SPLUNC1), now designated bactericidal/permeability-increasing protein fold-containing family A member 1 (BPIFA1), is an abundant secretory protein expressed primarily in the respiratory epithelium and released into airway surface liquid. SPLUNC1/BPIFA1 is a member of the BPI-fold protein family and exhibits several properties that support pulmonary homeostasis and mucosal defense [19,20]. It regulates airway surface liquid volume by protecting the epithelial sodium channel from proteolytic activation, reduces surface tension in airway secretions, and inhibits bacterial biofilm formation [21,22]. SPLUNC1/BPIFA1 also possesses direct antimicrobial activity and contributes to host defense against clinically relevant respiratory pathogens [21,22,23]. SPLUNC1 also regulates neutrophil recruitment and facilitates the resolution of inflammation. Mice lacking SPLUNC1/BPIFA1 exhibit increased susceptibility to pulmonary infection with *Klebsiella pneumoniae* and *Pseudomonas aeruginosa*, as well as impaired restriction of influenza A virus infection [23,24,25]. Collectively, these observations establish SPLUNC1/BPIFA1 as a multifunctional component of the airway epithelial defense system.

In addition to its antimicrobial functions, SPLUNC1/BPIFA1 has increasingly been implicated in the regulation of airway inflammation. SPLUNC1/BPIFA1 modulates epithelial inflammatory responses to microbial stimuli, and its deficiency enhances eosinophilic inflammation in experimental allergic airway disease [26,27,28]. SPLUNC1/BPIFA1 shares sequence homology with several LPS-binding proteins, including LBP (LPS Binding Protein) and BPI (Bactericidal/Permeability-Increasing Protein), and it has been proven that SPLUNC1 can bind to LPS in vitro [29]. These findings suggest that SPLUNC1/BPIFA1 may serve as an interface between epithelial sensing and leukocyte-mediated inflammation. However, its role in endotoxin-induced pulmonary inflammation remains unresolved, and previous studies have produced apparently divergent results. In a systemic model, intraperitoneal administration of LPS caused more severe lung injury and increased inflammatory cytokine expression in SPLUNC1-deficient mice, accompanied by altered accumulation of splenic CD11b^+^Gr-1^+^ myeloid cells [30]. In contrast, a separate study using 5 μg LPS delivered intranasally reported reduced pulmonary neutrophil recruitment and less peribronchial and alveolar inflammation in *Bpifa1*-deficient mice. That phenotype was associated with decreased CXCL9/CXCL10 expression and impaired interferon-dependent transcriptional responses [31]. These contrasting observations suggest that SPLUNC1/BPIFA1 may exert context-dependent effects on inflammation and underscore the need to define how its absence affects local immune-cell composition, cytokine production, tissue injury, and coordinated transcriptional programs within the lung.

In the present study, we used an integrated cellular and transcriptomic approach to determine how SPLUNC1/BPIFA1 deficiency influences the early pulmonary response to airway-delivered LPS. Wild-type (WT) and SPLUNC1/BPIFA1-knockout (KO) mice were challenged intranasally with 5 μg LPS and evaluated 24 h later using bronchoalveolar lavage, histopathology, cytospin differential analysis, flow cytometry, quantitative PCR, ELISA, and whole-lung RNA sequencing. We found that SPLUNC1/BPIFA1 deficiency resulted in increased inflammatory-cell recruitment, neutrophil-dominant airway inflammation, enhanced cytokine and chemokine production, and more severe lung injury. Transcriptomic analyses further revealed coordinated amplification of TNFα-NF-κB, IL-6-JAK-STAT3, complement, myeloid/neutrophil, and stress-response programs. Together, these findings identify SPLUNC1/BPIFA1 as an epithelial-derived regulator that restrains excessive inflammatory activation and protects the lung against endotoxin-induced injury.

## 2. Materials and Methods

### 2.1. SPLUNC1/BPIFA1 KO Mice and Animal Husbandry

SPLUNC1^−^/^−^ mice were originally generated through the screening of an ENU-induced T-to-A nonsense mutation in exon 2 of *Splunc1* on a C3HeB/FeJ background as previously described [23,24]. Homozygous wild-type (SPLUNC1^+/+^) littermates and knockout (SPLUNC1^−/−^) mice with the same genetic background were maintained for experimental use.

Male and female mice between 6 and 8 weeks old were maintained under specific pathogen-free conditions with 12 h of light/dark cycles and provided food and water ad libitum. All the mice were housed in an American Association for Review of Laboratory Animal Care-accredited animal facility. Protocols and studies involving animals were performed in accordance with the National Institutes of Health guidelines and were approved by the Institutional Animal Care and Use Committee of the University of Pittsburgh, protocol number 23083347.

### 2.2. LPS Lung Injury

A total of 57 mice were used in this study. Animals were randomly assigned to PBS (control group) or LPS treatment groups (For wild-type mice, 11 mice for PBS group, 13 mice for LPS group; for knockout mice, 14 mice for PBS group, 19 mice for LPS group). For LPS group, 5 µg LPS (from *Pseudomonas aeruginosa* 10, Sigma-Aldrich, St. Louis, MO, USA; L9143) dissolved in 50 µL PBS was administered intranasally (i.n.) under isoflurane anesthesia. Control group mice received an equal volume of PBS for comparison. Twenty-four hours after treatment, mice were euthanized, and samples were collected for bronchoalveolar lavage fluid (BALF), RNA extraction, protein analysis, flow cytometry, and histology analysis.

### 2.3. Bronchoalveolar Lavage and Cell Differential Counts

Twenty-four hours after PBS or LPS administration, mice were anesthetized with ketamine (80 mg/kg) and xylazine (8 mg/kg). The left lung was ligated and collected for RNA and protein extraction, and stored at −80 °C. The right lung was lavaged with 5 mL sterile PBS to collect BALF. The lavage procedure of mouse lungs was conducted as described [32].

One volume of BALF samples was mixed with 10 volumes red blood cell lysis buffer (Thermo fisher Scientific, Waltham, MA, USA, J62150.AP), vortex for 5 s, and incubate for 10 min at room temperature. Add 2 mL PBS to stop the reaction. Centrifuge at 300× *g* for 10 min at 4 °C. Resuspend cell pellets in PBS. Total cell numbers were determined using a Vision Cell Analyzer (Nexcelom, Lawrence, MA, USA) after staining with acridine orange. For differential cell counts, 2 × 10^4^ cells were resuspended in 200 μL PBS, deposited onto glass slides using a Cytospin^TM^ 4 cytocentrifuge (Epredia, Kalamazoo, MI, USA), and stained with Hemacolor Rapid staining kit (Sigma-Aldrich, St. Louis, MO, USA). Slides were examined using a Nikon Eclipse 90i microscope (Nikon Instruments Inc. Melville, NY, USA). A minimum of 400 cells was counted per slide to determine the percentages of macrophages, neutrophils, lymphocytes, and eosinophils.

### 2.4. Histology

The right lungs were inflated with 4% paraformaldehyde (PFA) and fixed at 4 °C for 8 h. The lungs were then transferred to PBS until paraffin embedding. Lung tissues were sectioned at 4 μm and stained with hematoxylin and eosin (H&E) following standard protocols. Histological changes, including inflammatory cell infiltration, alveolar septal thickening, edema, hemorrhage, and alveolar collapse, were evaluated. Lung injury was then graded using a four-point semiquantitative scoring system based on the overall severity of tissue damage. Briefly, a score of 0 indicated normal lung architecture with thin alveolar septa and well-defined respiratory bronchioles, alveolar sacs, and alveoli. A score of 1 indicated mild thickening of the alveolar septa with a slight reduction in alveolar air space. A score of 2 indicated diffuse alveolar septal thickening accompanied by inflammatory cell infiltration and a marked reduction in alveolar space. A score of 3 indicated widespread destruction of normal lung architecture [33]. Lung injury was scored independently by two investigators blinded to the treatment groups.

### 2.5. Flow Cytometry

BALF cells were centrifuged and washed once with PBS. Cell viability was assessed using Zombie Aqua viability dye according to the manufacturer’s instructions. After washing, cells were stained with fluorochrome-conjugated antibodies against Ly6G and CD45 for 30 min at 4 °C in the dark. Cells were then washed, fixed, and permeabilized before intracellular staining with antibodies against iNOS and CD206. Following a final wash, cells were resuspended in flow cytometry buffer and analyzed using a NovoCyte flow cytometer (Agilent Technologies, Santa Clara, CA, USA). Data were analyzed using NovoExpress software, version 1.5.1 (Agilent Technologies, Santa Clara, CA, USA).

Debris was excluded based on forward scatter (FSC) and side scatter (SSC), followed by doublet exclusion using FSC-A and FSC-H. Dead cells were excluded using Zombie Aqua staining. CD45^+^Ly6G^+^ cells were identified as neutrophils, and CD45^+^Ly6G^+^iNOS^+^ cells were defined as N1-like neutrophils. In Ly-6G^+^ cells, iNOS^−^ and CD 206^+^ cells were defined as N2-like neutrophils (Supplementary Figure S1).

### 2.6. RNA Extraction and qPCR Process

Total RNA was extracted from frozen lung tissues using TRIzol Reagent (Invitrogen, Thermo Fisher Scientific, Waltham, MA, USA) according to the manufacturer’s instructions. RNA concentration and purity were determined spectrophotometrically before reverse transcription. Equal amounts of RNA were reverse-transcribed into complementary DNA (cDNA) using a commercial reverse transcription kit according to the standard protocol [34].

Quantitative real-time PCR (qPCR) was performed using SYBR Green Master Mix on an Applied Biosystems QuantStudio^TM^ Pro 6 Real-Time PCR System (Thermo Fisher Scientific). Relative gene expression was calculated using the 2^−ΔΔCt^ method with *Actb* as the internal reference gene. Primer sequences used in this study are listed in Supplementary Table S1.

### 2.7. Protein Extraction and ELISA

Frozen lung tissues were homogenized in RIPA lysis buffer supplemented with a protease inhibitor cocktail (Thermo Fisher Scientific). Tissue lysates were centrifuged, and the supernatants were collected for protein analysis. Total protein concentration was determined using a BCA Protein Assay Kit (Thermo Fisher Scientific).

Protein levels of IL-1β and IL-6 were measured using DuoSet ELISA Development Kits (R&D Systems, Minneapolis, MN, USA) according to the manufacturer’s instructions. Cytokine concentrations were normalized to the total protein concentration of each sample and expressed as pg/mg total protein. All samples and standards were analyzed in duplicate.

### 2.8. RNA Sequencing

Total RNA was isolated from upper left lung lobes via TRIzol. RNA sequencing was performed by Dante Labs (L’Aquila, Italy) using a commercial bulk RNA sequencing service.

Raw sequencing data were imported into CLC Genomics Workbench for quality assessment [35], read processing, and alignment to the mouse reference genome (GRCm39/mm39). Gene expression values and differential expression results were generated using the CLC Genomics Workbench pipeline and exported for downstream analysis.

Downstream analyses were performed in R (version 4.5.2). Heatmaps, volcano plots, MA plots, and module score analyses were generated using normalized gene expression data. Kyoto Encyclopedia of Genes and Genomes (KEGG) pathway enrichment and Gene Set Enrichment Analysis (GSEA) were performed to identify biological pathways associated with SPLUNC1 deficiency following LPS exposure. Normalized enrichment scores (NES) and adjusted *p* values were used to assess pathway enrichment. Figures were generated using R packages appropriate for each analysis.

### 2.9. Statistical Analysis

Data are presented as mean ± SEM. Statistical analyses were performed using GraphPad Prism 11 (GraphPad Software, San Diego, CA, USA). Comparisons involving genotype and treatment were analyzed using two-way analysis of variance (ANOVA) followed by Tukey’s multiple-comparisons test. When only two groups were compared, an unpaired two-tailed Student’s *t* test was used. Welch’s *t* test was used for comparisons in which equal variances were not assumed. Bioinformatic analyses and data visualization were performed using R version 4.5.2. The value of *p* < 0.05 was considered statistically significant.

### 2.10. Software

Bioinformatic analyses were performed using CLC Genomics Workbench v23.0.5 (QIAGEN, Aarhus, Denmark) and R version 4.5.2. Statistical analyses were conducted using GraphPad Prism 11 (GraphPad Software, San Diego, CA, USA). Flow cytometry data were analyzed using NovoExpress version 1.5.1 (Agilent Technologies, Santa Clara, CA, USA).

## 3. Results

### 3.1. SPLUNC1/BPIFA1 Deficiency Exacerbates LPS-Induced Pulmonary Inflammation and Injury

**Figure 1:**
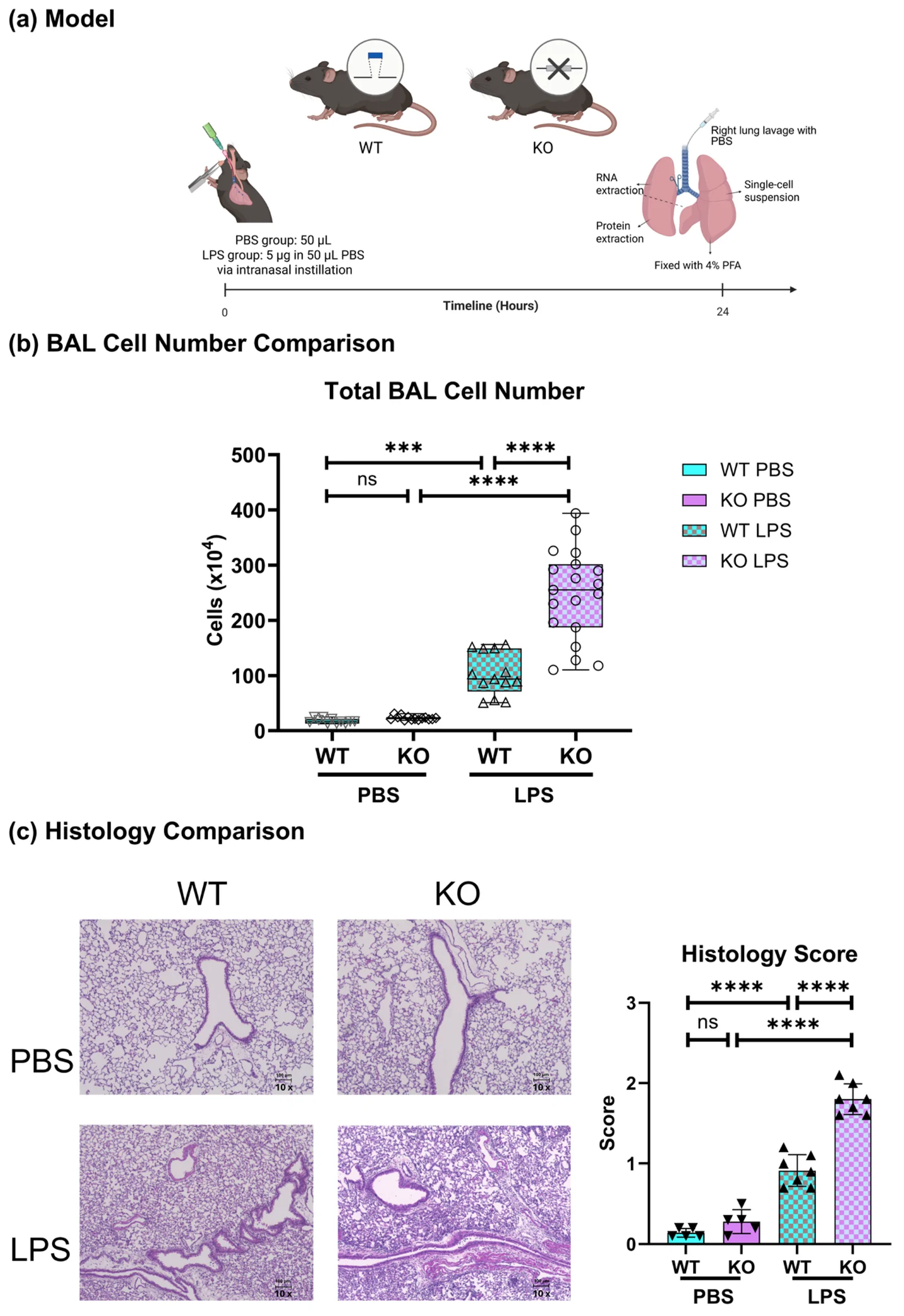
SPLUNC1/BPIFA1 deficiency increases inflammatory cell recruitment and lung injury following LPS challenge. (**a**) Experimental design. Wild-type (WT) and SPLUNC1/BPIFA1-knockout (KO) mice received 50 μL PBS or 5 μg lipopolysaccharides (LPS) in 50 μL PBS by intranasal instillation. At 24 h, bronchoalveolar lavage (BAL) was collected, and lungs were processed for single-cell analysis, histologic fixation with 4% paraformaldehyde, RNA extraction, and protein extraction. Created in BioRender. Lu, H. (2026) https://BioRender.com/bsphn5o. (**b**) Total BAL cell numbers in PBS- and LPS-treated WT and KO mice. (**c**) Representative lung histology from each group and quantitative histologic injury scores. Images were acquired at 10× magnification; scale bars, 100 μm. Each symbol represents an individual mouse. Data are presented as mean ± SEM; box plots indicate median, interquartile range, and minimum-to-maximum values. *n* = 5 mice per PBS group, 7 mice per LPS group. Statistical significance was determined using two-way ANOVA followed by Tukey multiple comparison test. *** *p* < 0.001 and **** *p* < 0.0001; ns, not significant.

To determine whether SPLUNC1/BPIFA1 regulates the pulmonary response to endotoxin, wild-type (WT) and SPLUNC1/BPIFA1-knockout (KO) mice received intranasal PBS or 5 μg LPS, and lungs were evaluated 24 h later (Figure 1a). Total bronchoalveolar lavage (BAL) cell numbers were comparable between PBS-treated WT and KO mice. LPS challenge increased BAL cellularity in both genotypes; however, the increase was substantially greater in SPLUNC1 KO mice than in WT mice (*p* < 0.0001; Figure 1b).

Consistent with the BAL findings, PBS-treated WT and KO mice exhibited largely preserved lung architecture and similar histology scores. LPS induced inflammatory cell accumulation and alveolar septal thickening in WT lungs, whereas KO lungs showed more extensive inflammatory infiltration and tissue disruption. Quantitative histologic scoring confirmed significantly greater lung injury in LPS-treated KO mice than in LPS-treated WT mice (*p* < 0.0001; Figure 1c). Thus, loss of SPLUNC1/BPIFA1 increased both inflammatory cell recruitment and the severity of acute lung injury following LPS exposure.

### 3.2. SPLUNC1/BPIFA1 Deficiency Promotes Neutrophil-Dominant Airway Inflammation

**Figure 2:**
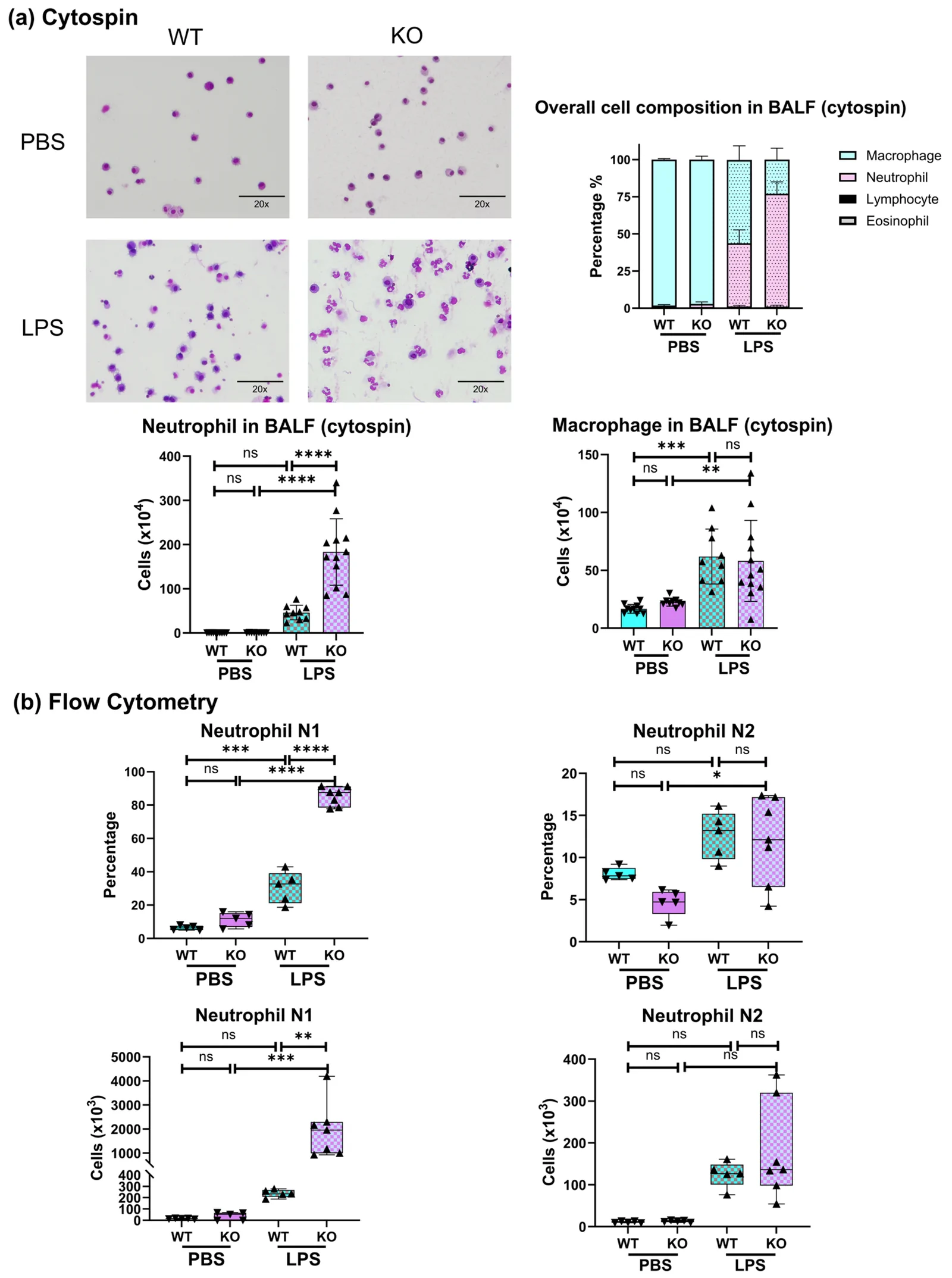
SPLUNC1/BPIFA1 deficiency promotes neutrophil-dominant airway inflammation following LPS challenge. (**a**) Representative cytospin preparations of BAL cells collected 24 h after intranasal administration of PBS or LPS to WT and SPLUNC1/BPIFA1-KO mice. Images were acquired at 20× magnification; scale bars, 100 μm. The stacked bar graph shows the relative proportions of macrophages, neutrophils, lymphocytes, and eosinophils in BAL. Absolute neutrophil and macrophage numbers were calculated from total BAL cell counts and differential cell percentages. (**b**) Flow-cytometric quantification of neutrophil populations in BAL. Results are shown as the percentage and absolute number of cells within the N1 and N2 neutrophil gates. Flow-defined populations were identified as follows: N1, [CD45^+^Ly6G^+^iNOS^+^]; N2, [CD45^+^Ly6G^+^iNOS^−^CD206^+^]. Each symbol represents an individual mouse. Data are presented as mean ± SEM; box plots indicate median, interquartile range, and minimum-to-maximum values. *n* = 10 for WT PBS, *n* = 9 for KO PBS, *n* = 9 for WT LPS, *n* = 12 for KO LPS for cytospin. *n* = 5 for PBS groups and WT LPS group, *n* = 8 for KO LPS group mice for flow cytometry. Statistical significance was determined using two-way ANOVA followed by Tukey multiple-comparisons test. * *p* < 0.05, ** *p* < 0.01, *** *p* < 0.001, and **** *p* < 0.0001; ns, not significant.

Differential analysis of BAL cells demonstrated that the increased cellularity in LPS-treated KO mice was driven predominantly by neutrophil recruitment. Cytospin preparations from PBS-treated mice consisted primarily of macrophages, with few neutrophils in either genotype. LPS challenge caused a marked shift toward neutrophilic inflammation, which was substantially more pronounced in KO mice (Figure 2a). Both the relative abundance and absolute number of BAL neutrophils were significantly greater in LPS-treated KO mice than in LPS-treated WT mice (*p* < 0.0001). In contrast, total macrophage numbers did not differ significantly between the two LPS-treated genotypes.

Flow cytometry analysis further characterized the inflammatory cell populations (Figure 2b). To distinguish changes in neutrophil phenotype from changes in overall recruitment, we examined N1-like and N2-like populations. N1-like neutrophils are generally associated with pro-inflammatory activity, whereas N2-like neutrophils are more closely linked to anti-inflammatory and tissue repair functions [36,37,38]. In this study, N1-like neutrophils were defined as CD45^+^Ly6G^+^iNOS^+^ cells. N2-like neutrophils were defined as CD45^+^Ly6G^+^iNOS^−^CD206^+^ cells (Supplementary Figure S1). LPS increased the proportion of cells within the N1 neutrophil gate in both genotypes, but this population was markedly expanded in KO mice compared with WT mice (*p* < 0.0001). The absolute number of N1 neutrophils was also significantly greater in LPS-treated KO mice (*p* < 0.0001). In contrast, the absolute number of cells within the N2 neutrophil gate did not differ significantly between LPS-treated WT and KO mice.

Collectively, these findings demonstrate that SPLUNC1/BPIFA1 deficiency produces a profound shift in the airway inflammatory-cell landscape, characterized by preferential expansion of the N1 neutrophil population.

### 3.3. SPLUNC1/BPIFA1 Deficiency Amplifies Inflammatory Cytokine and Chemokine Production

**Figure 3:**
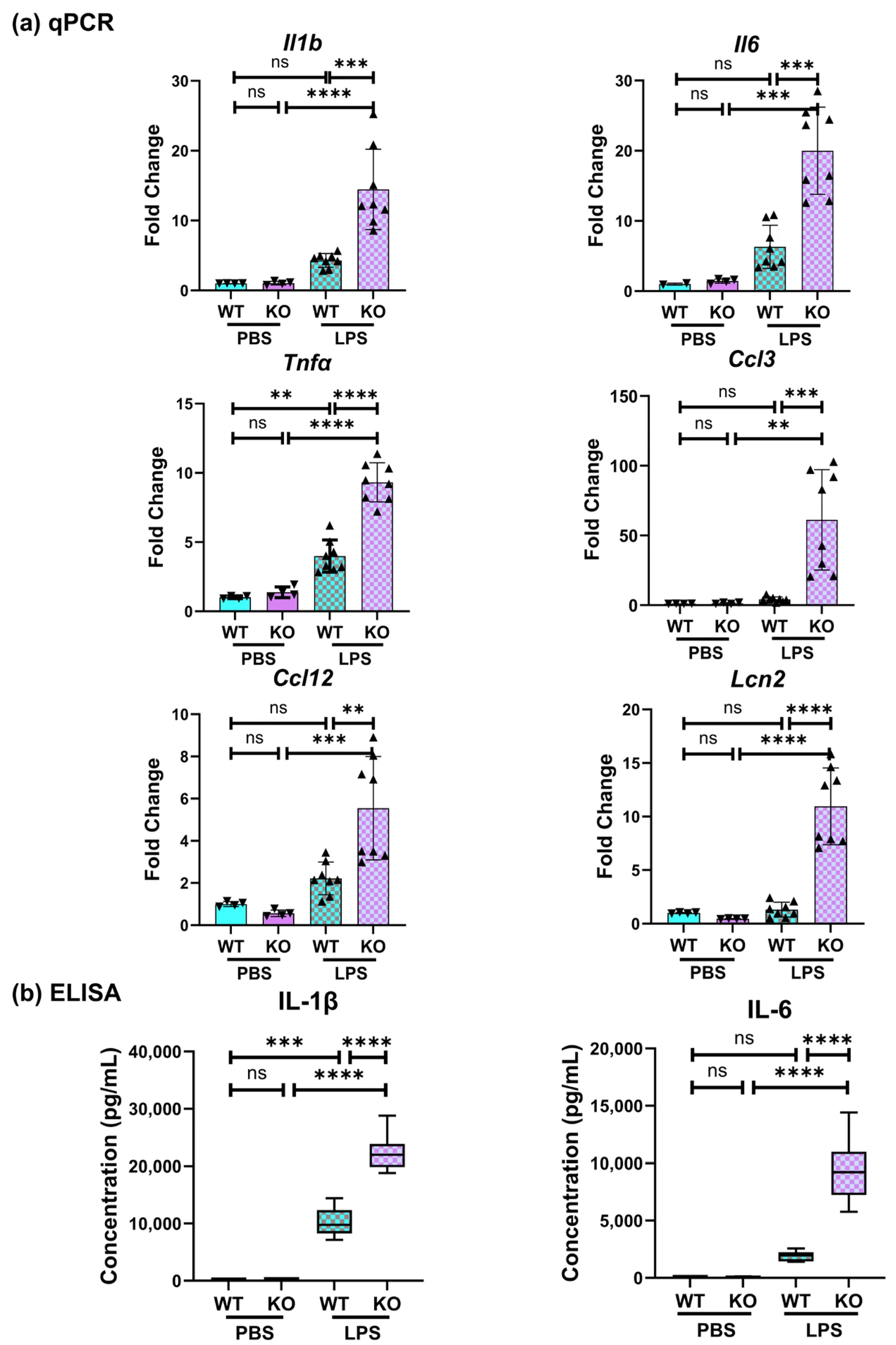
SPLUNC1/BPIFA1 deficiency amplifies inflammatory cytokine and chemokine expression following LPS challenge. (**a**) Whole-lung mRNA expression of *Il1b*, *Il6*, *Tnf*, *Ccl3*, *Ccl12*, and *Lcn2* was measured by quantitative real-time PCR 24 h after intranasal administration of PBS or LPS to WT and SPLUNC1/BPIFA1-KO mice. Transcript levels were normalized to WT PBS and calculated using the 2^−ΔΔCt^ method relative to WT PBS. (**b**) IL-1β and IL-6 protein concentrations in lung homogenates were measured by ELISA. Each symbol represents an individual mouse. Data are presented as mean ± SEM; box plots indicate median, interquartile range, and minimum-to-maximum values. *n* = 4 mice per PBS group, 8 mice per LPS group for quantitative PCR and 4 mice per PBS group, 8 mice per LPS group for ELISA. Statistical significance was determined using Two-way ANOVA followed by Tukey multiple comparison. ** *p* < 0.01, *** *p* < 0.001, and **** *p* < 0.0001; ns, not significant.

We next examined whether the increased inflammatory-cell recruitment in KO mice was accompanied by enhanced expression of inflammatory mediators. Under PBS-treated conditions, whole-lung mRNA levels of *Il1b*, *Il6*, *Tnf*, *Ccl3*, *Ccl12*, and *Lcn2* were generally low and did not differ significantly between WT and KO mice (Figure 3a). LPS challenge increased the expression of these genes, but the magnitude of induction was consistently greater in KO lungs. Compared with LPS-treated WT mice, LPS-treated KO mice exhibited significantly higher expression of *Il1b* (*p* < 0.001), *Il6* (*p* < 0.001), *Tnf* (*p* < 0.0001), *Ccl3* (*p* < 0.001), *Ccl12* (*p* < 0.01), and *Lcn2* (*p* < 0.0001).

The enhanced transcriptional response was confirmed at the protein level. LPS induced the production of IL-1β and IL-6 in both genotypes, but the concentrations of both cytokines were significantly higher in lung protein extracts from KO mice than from WT mice (*p* < 0.0001; Figure 3b). These results indicate that SPLUNC1/BPIFA1 deficiency broadly enhances the pulmonary cytokine and chemokine response to LPS rather than selectively affecting a single inflammatory mediator.

### 3.4. RNA Sequencing Reveals Enhanced Inflammatory Transcriptional Pathway Activation in SPLUNC1/BPIFA1-Deficient Lungs

**Figure 4:**
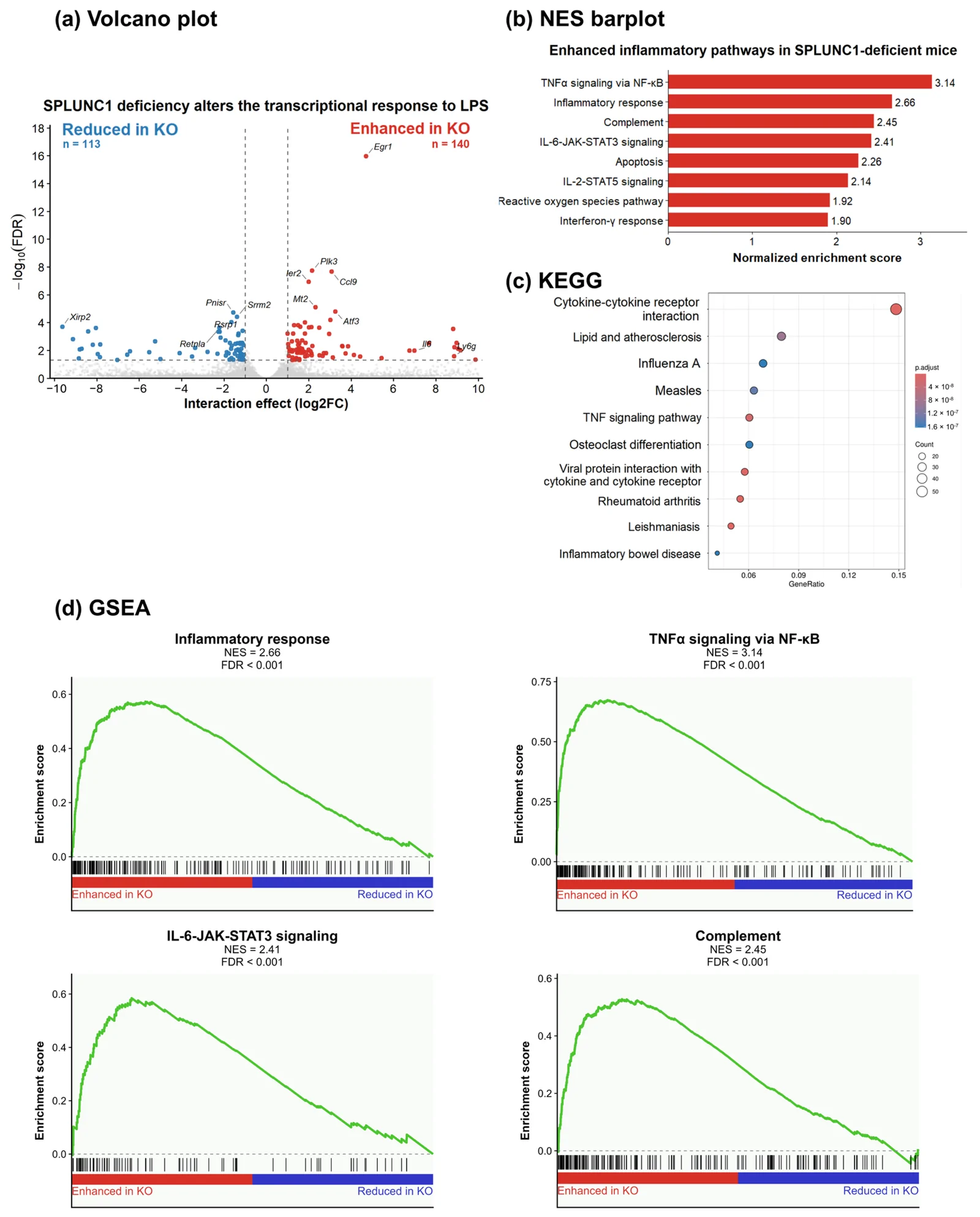
RNA sequencing identifies enhanced inflammatory transcriptional responses in SPLUNC1/BPIFA1-deficient lungs. Whole-lung RNA sequencing was performed 24 h after PBS or LPS challenge in WT and SPLUNC1/BPIFA1-KO mice. (**a**) Volcano plot showing the genotype-by-treatment interaction effect on the transcriptional response to LPS. The *x*-axis represents the interaction effect expressed as log2 fold change, and the *y*-axis represents −log10 of the false-discovery rate (FDR). Red symbols indicate genes with enhanced LPS responses in KO mice (*n* = 140), whereas blue symbols indicate genes with reduced responses in KO mice (*n* = 113). Colored genes met the prespecified criteria of |log2 fold change| > 1 and FDR < 0.05. The vertical dashed lines indicate |log2 fold change| > 1, and the horizontal dashed line indicates FDR = 0.05. (**b**) Normalized enrichment scores (NES) for inflammatory Hallmark gene sets preferentially enriched in SPLUNC1/BPIFA1-KO lungs. (**c**) KEGG pathway enrichment analysis of genes exhibiting genotype-dependent responses to LPS. Symbol size represents the number of genes assigned to each pathway, and color represents the adjusted *p* value. (**d**) Gene-set enrichment analysis plots for the inflammatory response (NES = 2.66), TNFα/NF-κB signaling (NES = 3.14), IL-6-JAK-STAT3 signaling (NES = 2.41), and complement activation (NES = 2.45). All four gene sets were enriched toward genes with enhanced responses in KO mice (FDR < 0.001). *n* = 2 mice per PBS group, *n* = 3 for WT LPS group, *n* = 4 for KO LPS group.

To define the broader transcriptional consequences of SPLUNC1/BPIFA1 deficiency, we performed RNA sequencing of lungs from PBS- and LPS-treated WT and KO mice. Genotype-by-treatment interaction analysis identified 253 genes whose significant responses to LPS differed between genotypes, including 140 upregulated and 113 downregulated genes in SPLUNC1-deficient lungs compared with WT controls (Figure 4a). Among the most strongly upregulated genes were several inflammation-associated transcripts, including *Egr1*, *Il6*, *Ly6g*, *Ccl9*, *Ier2*, *Atf3*, and *Mt2*, whereas relatively few genes showed marked downregulation. Thus, SPLUNC1/BPIFA1 deficiency substantially remodeled the lung transcriptional response to LPS.

To determine whether these transcriptional changes were associated with specific biological pathways, enrichment analyses were performed. Hallmark gene set analysis demonstrated preferential activation of multiple inflammatory pathways in SPLUNC1-deficient mouse lungs (Figure 4b). TNFα signaling through NF-κB showed the strongest enrichment (normalized enrichment score [NES] = 3.14), followed by the inflammatory response (NES = 2.66), complement (NES = 2.45), IL6-JAK-STAT3 signaling (NES = 2.41), apoptosis (NES = 2.26), IL2-STAT5 signaling (NES = 2.14).

KEGG pathway analysis similarly identified enrichment of cytokine-cytokine receptor interactions, TNF signaling, and multiple immune- and inflammation-associated pathways (Figure 4c). Consistent with these results, GSEA plots confirmed significant enrichment of the inflammatory response, TNF-NF-κB signaling, IL6-JAK-STAT3 signaling, and complement sets toward genes enhanced in SPLUNC1/BPIFA1 knockout mice (FDR < 0.001 for each pathway; Figure 4d). These transcriptomic findings are consistent with the increased cytokine expression, neutrophil recruitment, and lung injury observed in SPLUNC1/BPIFA1-deficient mice.

### 3.5. SPLUNC1/BPIFA1 Deficiency Coordinately Enhances Multiple Inflammatory Gene Programs

**Figure 5:**
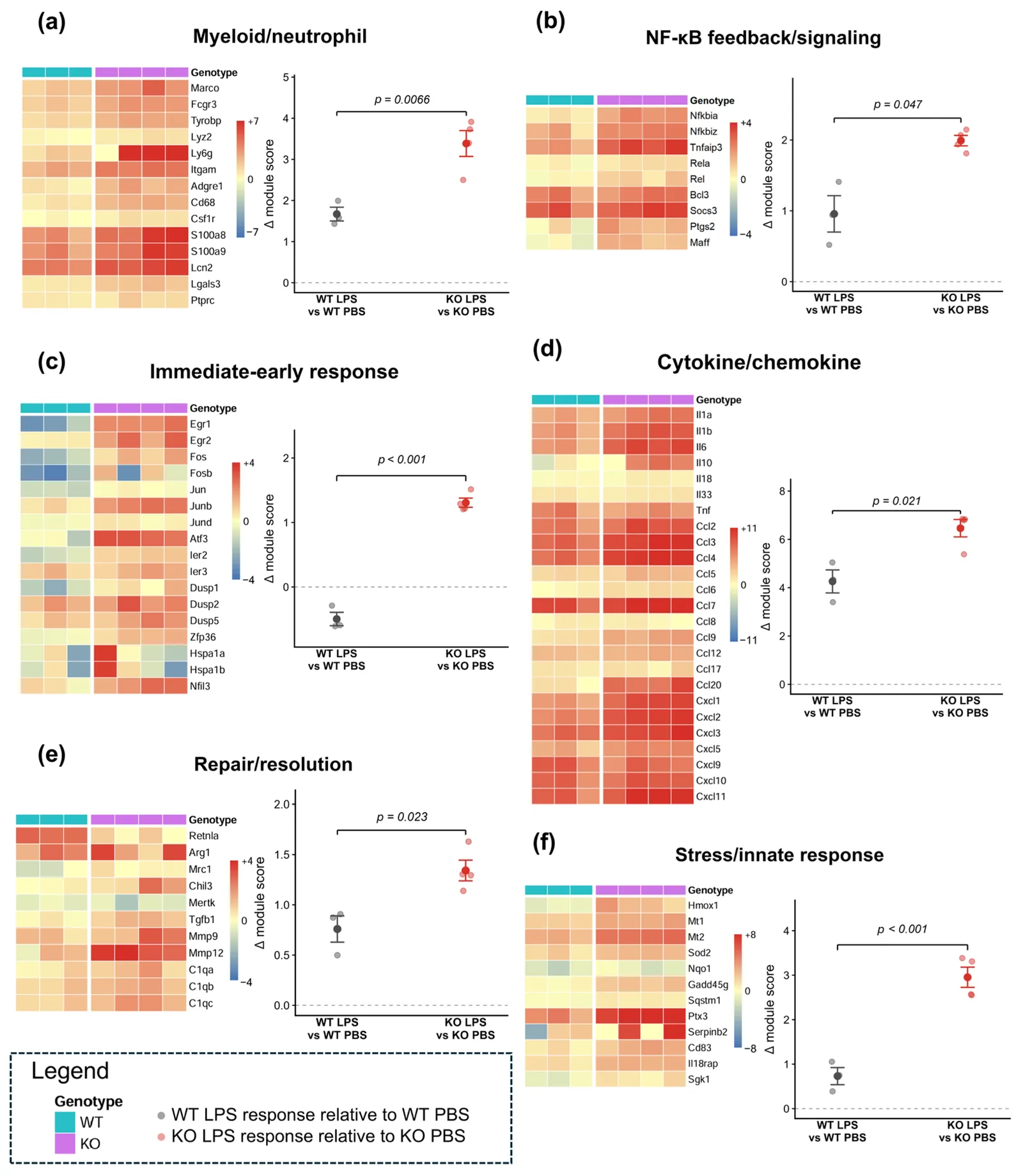
SPLUNC1/BPIFA1 deficiency amplifies coordinated inflammatory gene programs following LPS challenge. (**a**–**f**) Genotype-matched PBS-corrected heatmaps and corresponding module scores for six LPS-responsive gene programs: (**a**) myeloid/neutrophil, (**b**) NF-κB feedback/signaling, (**c**) immediate-early response, (**d**) cytokine/chemokine, (**e**) repair/resolution, and (**f**) stress/innate response. Heatmaps show LPS-induced changes in gene expression relative to the corresponding genotype-matched PBS controls. Values represent changes in log2-transformed DESeq2-normalized counts relative to the genotype-matched PBS mean; red indicates increased and blue indicates decreased expression relative to PBS. Module scores were calculated from log2-transformed DESeq2-normalized counts and expressed relative to genotype-matched PBS controls. SPLUNC1/BPIFA1 deficiency was associated with significantly higher module scores for myeloid/neutrophil (*p* = 0.0066), NF-κB feedback/signaling (*p* = 0.047), immediate-early response (*p* < 0.001), cytokine/chemokine (*p* = 0.021), repair/resolution (*p* = 0.023), and stress/innate response (*p* < 0.001). Small symbols represent individual biological replicates, and large symbols with error bars indicate mean ± SEM. *n* = 3 WT-LPS and *n* = 4 KO-LPS mice. *p* values were determined using unpaired two-tailed Welch’s *t*-tests comparing genotype-matched PBS-corrected LPS responses between WT and KO mice.

To examine the organization of the LPS response, representative inflammatory genes were grouped into six biologically related functional modules, including myeloid and neutrophil responses, NF-κB feedback signaling, immediate-early response, cytokine and chemokine production, tissue repair and resolution, and stress and innate immune responses (Figure 5a–f). Heatmap analysis demonstrated greater LPS-induced activation of these coordinated gene programs in KO lungs than in WT lungs. To better compare the transcriptional response to LPS between genotypes, calculation of the LPS-induced change relative to the genotype-matched PBS group further showed that many cytokine/chemokine and myeloid/neutrophil genes underwent stronger induction in KO mice (Figure 5a,d).

Although LPS induced inflammatory gene expression in both WT and SPLUNC1-deficient lungs, PBS-normalized analysis showed that the transcriptional response to LPS was consistently stronger in KO mice. Genes involved in the immediate-early response, including *Egr1*, *Fos*, *Jun*, *Atf3*, and *Ier2*, were more strongly induced in KO mice (Figure 5c). A similar pattern was observed for genes associated with NF-κB signaling, including *Nfkbia*, *Nfkbiz*, *Tnfaip3*, *Rel*, and *Socs3* (Figure 5b). Expression of multiple cytokines and chemokines were also consistently higher in SPLUNC1/BPIFA1-deficient mouse lungs (Figure 5d).

Myeloid/neutrophil-associated genes, including *Ly6g*, *S100a8*, *S100a9*, *Lcn2*, and *Lgals3*, were more strongly induced in KO mice (Figure 5a). Enhanced responses were also observed among stress/innate-response genes, including *Hmox1*, *Mt1*, *Mt2*, *Sod2*, *Nqo1*, *Gadd45g*, and *Ptx3* (Figure 5f). Repair/resolution-associated genes, including *Arg1*, *Mrc1*, *Chil3*, *Mertk*, *Tgfb1*, *Mmp9*, and *Mmp12*, also showed higher overall responses in KO lungs, although the magnitude of change was more modest than that observed for several inflammatory modules (Figure 5e). Module score analysis confirmed that all six programs were significantly more strongly activated in KO lungs. These included myeloid/neutrophil activation (*p* = 0.0066), NF-κB feedback/signaling (*p* = 0.047), immediate-early response (*p* < 0.001), cytokine/chemokine production (*p* = 0.021), repair/resolution (*p* = 0.023), and stress/innate response (*p* < 0.001). Therefore, SPLUNC1/BPIFA1 deficiency did not simply increase individual cytokines but amplified a coordinated network encompassing inflammatory signaling, neutrophil recruitment, cellular stress, and compensatory tissue-response pathways.

Together, these findings identify SPLUNC1/BPIFA1 as an important regulator of pulmonary inflammatory homeostasis. Its absence results in exaggerated inflammatory signaling, excessive neutrophil accumulation, and increased tissue injury following LPS challenge.

## 4. Discussion

The present study demonstrates that SPLUNC1/BPIFA1 deficiency markedly amplifies the pulmonary response to airway-delivered LPS. Using the exon-2 point-mutation mouse line originally described in the *Klebsiella pneumoniae* study, we found that loss of SPLUNC1/BPIFA1 increased BAL cellularity, promoted neutrophil-dominant inflammation, enhanced cytokine and chemokine production, and aggravated histologic lung injury. Whole-lung RNA sequencing extended these cellular observations by identifying coordinated activation of TNFα-NF-κB, IL-6-JAK-STAT3, complement, myeloid/neutrophil, oxidative-stress, and tissue-response pathways. These results support a model in which epithelial-derived SPLUNC1/BPIFA1 functions as a homeostatic regulator that limits excessive inflammatory amplification following endotoxin exposure.

The increased BAL cellularity in SPLUNC1/BPIFA1-deficient mice was driven primarily by neutrophil accumulation. Cytological differential cell count analysis demonstrated a pronounced shift from a macrophage-dominant BAL profile under basal conditions to a neutrophil-dominant profile after LPS challenge, with a substantially greater response in knockout mice. Flow cytometry further revealed marked expansion of the population defined by the N1 neutrophil gate, whereas absolute numbers within the N2 gate did not differ significantly between LPS-treated genotypes. Although the functional N1/N2 framework may help describe neutrophil heterogeneity, these designations should be interpreted cautiously because neutrophils occupy dynamic transcriptional and functional states that may not be fully captured by a limited surface-marker panel [39]. Nevertheless, the agreement between cytospin and flow-cytometric analyses provides strong evidence that exaggerated neutrophil recruitment is a major feature of the knockout phenotype. Neutrophils play an essential role in the early innate immune response by eliminating invading pathogens. However, excessive neutrophil recruitment can also amplify tissue injury by releasing reactive oxygen species, proteases, and inflammatory mediators. The increased expression of *Lcn2*, *Mpo*, and *S100a8/S100a9* observed in SPLUNC1/BPIFA1-deficient lungs is consistent with enhanced neutrophil activation and antimicrobial responses. These changes may contribute to the greater lung injury observed in KO mice following LPS challenge.

The exaggerated cellular response was accompanied by broad induction of inflammatory mediators. Compared with LPS-treated WT mice, knockout mice exhibited higher expression of *Il1b*, *Il6*, *Tnf*, *Ccl3*, *Ccl12*, and *Lcn2*, together with increased IL-1β and IL-6 protein concentrations. *IL-1β*, *IL-6*, and *TNFα* are central components of the acute inflammatory response and can promote endothelial and epithelial activation, leukocyte recruitment, and barrier dysfunction [40]. CCL3 and CCL12 further support myeloid-cell recruitment, whereas LCN2 is induced during epithelial stress and neutrophilic inflammation. The simultaneous enhancement of these mediators indicates that SPLUNC1/BPIFA1 deficiency does not simply alter one chemotactic pathway but instead produces a broadly heightened inflammatory state. This conclusion is consistent with previous evidence that SPLUNC1/BPIFA1 modulates epithelial and macrophage responses to microbial and allergic stimuli [26,27] and that its absence increases inflammation during bacterial infection [23,24].

The transcriptomic analyses provide a system-level context for these cellular and molecular findings. Genotype-by-treatment interaction analysis identified 140 genes with enhanced and 113 genes with attenuated responses to LPS in knockout lungs, demonstrating that SPLUNC1/BPIFA1 deficiency reshapes rather than uniformly increases the endotoxin response. Rather than affecting only a small number of inflammatory genes, SPLUNC1 deficiency altered multiple inflammatory pathways simultaneously. Hallmark and KEGG enrichment analyses consistently identified TNFα signaling through NF-κB as the strongest enrichment, accompanied by activation of the inflammatory-response, complement, IL-6-JAK-STAT3, apoptosis, reactive-oxygen-species, and interferon-γ programs [41,42,43,44]. These pathways provide plausible links between the cytokine measurements, neutrophil accumulation, and tissue injury [45,46]. In particular, enhanced NF-κB activity could account for the coordinated induction of IL1b, IL6, TNF, and multiple chemokines, while IL-6-JAK-STAT3 signaling may sustain inflammatory-cell activation and survival [47,48]. Module-level analysis reinforced the conclusion that the knockout phenotype represents a coordinated inflammatory state. Immediate-early, NF-κB feedback/signaling, cytokine/chemokine, myeloid/neutrophil, stress/innate-response, and repair/resolution modules were all more strongly induced in knockout lungs [49,50].

Our findings are consistent with the systemic LPS study by Zhang and colleagues, in which SPLUNC1-deficient mice developed greater lung inflammation and injury following intraperitoneal LPS administration [30]. However, they differ from the findings of Britto and colleagues, who reported reduced neutrophil recruitment, less peribronchial and alveolar inflammation, and diminished CXCL9/CXCL10 and interferon signaling in *Bpifa1*-deficient mice after intranasal administration of 5 μg LPS [31]. The divergent results cannot be explained simply by the route, dose, or 24 h endpoint because these parameters were comparable to those used in the present study. Instead, an important distinction is the genetic model. The mice used here carry an ENU-induced T-to-A nonsense mutation in exon 2 of Splunc1, producing an L50X premature stop codon on the C3HeB/FeJ background. SPLUNC1 protein is undetectable in the trachea and BAL of these mice, confirming a functional null phenotype [23,24]. In contrast, Britto and colleagues used a gene-ablating deletion generated in C57BL/6 embryonic stem cells [31].

LPS source and biochemical composition may be relevant; Britto and colleagues used LPS derived from *P. aeruginosa* PAO1, whereas differences in bacterial species, serotype, purification, or contaminating microbial components can alter the relative activation of TLR4-dependent signaling pathways. Additional variables-including age, sex, mouse strains, housing conditions, microbiome composition, and colony-specific immune status-could further modify the response. Finally, Britto’s study used Agilent-based G3 Mouse Gene Expression v.2 8 × 60K microarrays, while our studies employed mRNA-sequencing approaches. These possibilities should not be interpreted as invalidating either model. Rather, the combined findings suggest that SPLUNC1/BPIFA1 is not simply proinflammatory or anti-inflammatory but may act as a context-dependent regulator of the magnitude and composition of the pulmonary response.

One potential framework for reconciling the studies is that SPLUNC1/BPIFA1 differentially regulates the MyD88-NF-κB and TRIF-interferon branches of LPS signaling. Britto and colleagues proposed that SPLUNC1/BPIFA1 supports interferon-dependent CXCL10 production and neutrophil recruitment, because its deletion reduced CXCL9/CXCL10 and interferon-responsive genes [31]. In our model, the dominant consequence of SPLUNC1/BPIFA1 loss was instead enhanced TNFα-NF-κB, IL-6-JAK-STAT3, complement, and cytokine/chemokine activation. SPLUNC1/BPIFA1 may therefore support selected interferon-mediated host-defense responses while simultaneously restraining excessive NF-κB-centered inflammatory amplification. The balance between these functions could vary with genetic background, LPS structure, cellular context, or phase of the response [51]. This hypothesis is consistent with the ability of BPI-fold proteins to interact -with bacterial lipids and influence pattern-recognition signaling, but direct comparisons of TLR4 proximal signaling, MyD88/TRIF activation, and interferon responses will be required to test it [20,52]. The mechanism by which SPLUNC1/BPIFA1 affects the LPS response is still unclear. Direct binding to LPS has been reported in vitro [29]. However, SPLUNC1/BPIFA1 has also been linked to inflammatory signaling through other pathways, such as Orai1-mediated Ca^2+^ signaling [42,43]. Studies in isolated epithelial cells or macrophages will help determine whether these mechanisms contribute to the LPS response.

The present study should be interpreted in the context of some limitations. First, this study focused on the acute phase of LPS-induced lung injury, and all analyses were performed 24 h after LPS administration. The 24 h endpoint captures the established acute inflammatory phase but does not distinguish early initiation from later amplification or resolution. The role of SPLUNC1 during the resolution phase of inflammation and tissue repair remains to be determined. RNA sequencing was performed using whole-lung tissue. Although this approach identified coordinated inflammatory transcriptional programs, it could not distinguish cell type-specific responses [53,54]. Future studies using single-cell transcriptomics or cell-specific approaches may provide greater insight into the cellular mechanisms regulated by SPLUNC1/BPIFA1. In addition, this study identified enhanced activation of multiple inflammatory pathways in SPLUNC1-deficient lungs; however, the upstream mechanisms linking SPLUNC1 deficiency to these transcriptional changes remain to be defined. Additional mechanistic studies will be needed to determine how SPLUNC1/BPIFA1 regulates inflammatory signaling during acute lung injury. Because this study used a constitutive SPLUNC1/BPIFA1 knockout model, compensatory changes cannot be excluded. A rescue strategy with airway-delivered recombinant SPLUNC1/BPIFA1 would help determine whether restoring SPLUNC1 reverses the inflammatory phenotype.

## 5. Conclusions

Our findings demonstrate that SPLUNC1/BPIFA1 limits excessive pulmonary inflammation during acute lung injury. Loss of SPLUNC1/BPIFA1 amplifies LPS-induced inflammatory signaling, promotes neutrophil-dominant airway inflammation, and increases histologic lung injury. The coordinated activation of NF-κB, IL-6-JAK-STAT3, complement, myeloid/neutrophil, and stress-response programs identify SPLUNC1/BPIFA1 as an important regulator of pulmonary inflammatory homeostasis. These results support a protective role for SPLUNC1 in maintaining pulmonary immune homeostasis and provide a foundation for future studies investigating SPLUNC1 as a potential therapeutic target for acute inflammatory lung diseases.

## Data Availability

The original contributions presented in this study are included in the article/Supplementary Material. Further inquiries can be directed to the corresponding author.

## Supplementary Materials

The following supporting information can be downloaded at: https://www.mdpi.com/article/10.3390/biom16091349/s1, Table S1: Primer sequences used for quantitative real-time PCR. Figure S1: Representative gating strategy used for flow cytometric analysis of bronchoalveolar lavage fluid (BALF) cells.

## Abbreviations

The following abbreviations are used in this manuscript:

ALI	Acute lung injury
BALF	Bronchoalveolar lavage fluid
BPIFA1	Bactericidal/permeability-increasing protein fold-containing family A member 1
SPLUNC1	Short palate, lung, and nasal epithelial clone 1
LPS	Lipopolysaccharide
ELISA	Enzyme-linked immunosorbent assay
GSEA	Gene Set Enrichment Analysis
KEGG	Kyoto Encyclopedia of Genes and Genomes
qPCR	Quantitative real-time polymerase chain reaction
RNA-seq	RNA sequencing
WT	Wild Type
KO	Knockout
NF-κB	Nuclear factor kappa B
TLR4	Toll-like receptor 4
MyD88	Myeloid differentiation primary response 88
