# Supplementary material for: SPLUNC1/BPIFA1 Restrains Neutrophil-Dominant Inflammation and Coordinated Inflammatory Gene Programs During LPS-Induced Lung Injury
Source: Biomolecules. 2026 Sep 16;16(9):1349. doi: 10.3390/biom16091349 (PMC13604270; doi:10.3390/biom16091349)
Supplement: Supplementary file 1 [file biomolecules-16-01349-s001.zip › biomolecules-4543317-supplementary.pdf]

Table S1. Primer sequences used for quantitative real-time PCR.

Gene	Forward primer	Reverse primer
Il1b	GCAACTGTTCCTGAACTCAAC T	ATCTTTTGGGGTCCGTCAACT
Il6	TAGTCCTTCCTACCCCAATTTCC	TTGGTCCTTAGCCACTCCTTC
Tnf-α	CCCTCACACTCA GATCATCTTCT	GCTACGACGTGGGCTACAG
Ccl3	TTCTCTGTACCATGACACTCTGC	CGTGGAATCTTCCGGCTGTAG
Lcn2	GGCCAGTTCACT CTGGGAAA	TGGCGAACTGGT TGTAGTCC
Ccl12	GCTACAGGAGAATCACAAGCAGC	AC-<br>GTCTTATCCAAGTGGTTTATGG
Actb	GTGACGTTGACATCCGTAAAGA	GCCGGACTCATCGTACTCC

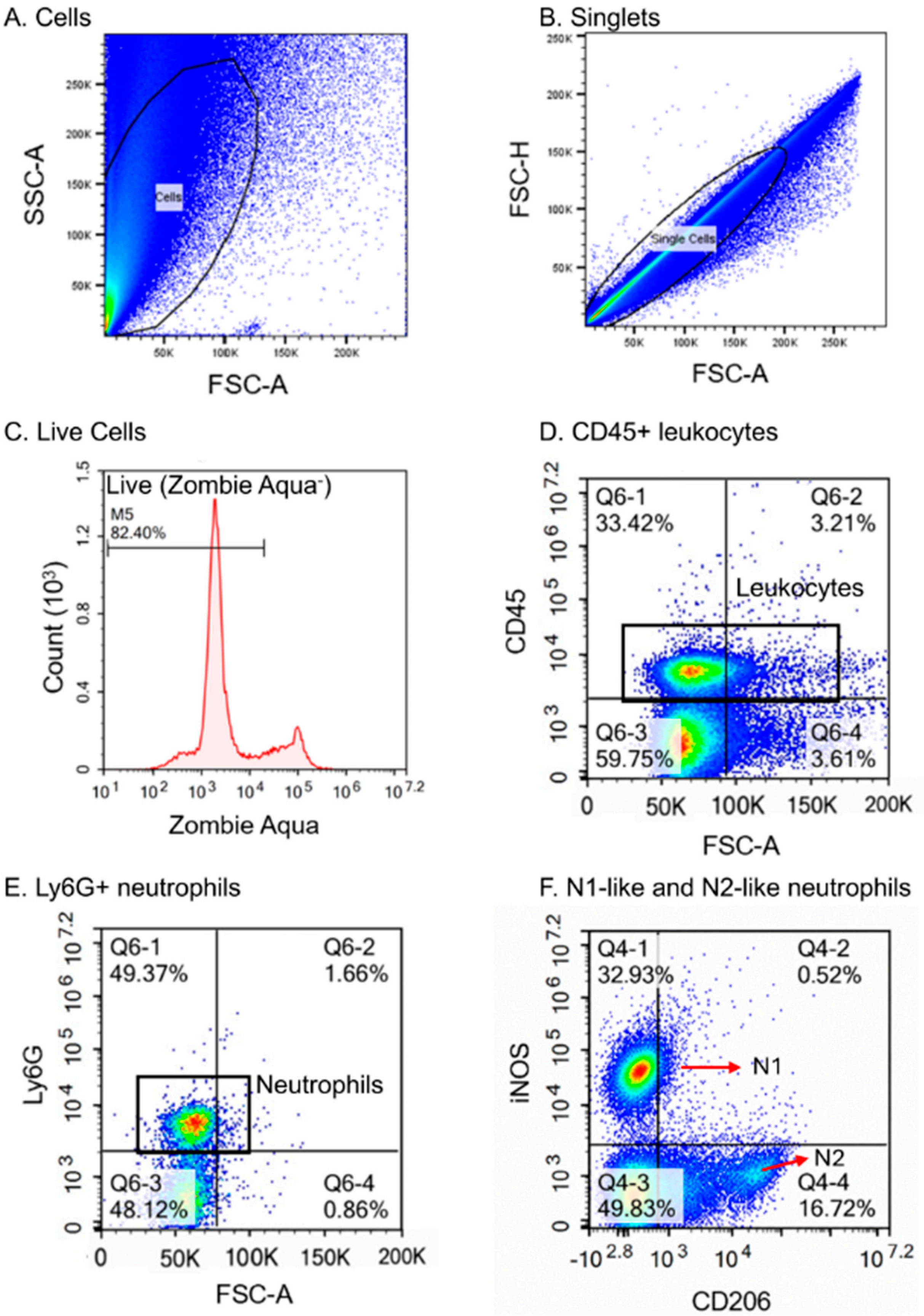

Figure S1. Representative gating strategy used for flow cytometric analysis of bronchoalveolar lavage fluid (BALF) cells. (A) Cells were initially gated based on forward scatter (FSC-A) and side scatter (SSC-A). (B) Singlets were selected using FSC-A and FSC-H. (C) Live cells were identified as Zombie Aquanegative cells. (D) CD45* leukocytes were gated from the live-cell population. (E)

Ly6G* neutrophils were subsequently identified within the CD45* population. (F) Expression of iNOS and CD206 was used to further characterize N1-like and N2-like neutrophil populations. The same gating strategy was applied consistently across all experimental groups.
